# Mesenteric Panniculitis and the Gut–Mesentery–Metabolic Axis: A Hypothesis-Generating Narrative Review

**DOI:** 10.3390/biomedicines14092017

**Published:** 2026-09-08

**Authors:** Sorina Ispas, Viviana Maggio, Syed Arman Rabbani, Adil Farooq Wali, Bhoomendra A. Bhongade, Sirajunisa Talath, Imran Rashid Rangraze, Shakta Mani Satyam, Ashot Avagimyan, Karolina Hoffmann, Ioannis Ilias, Anna Paczkowska, Mohamed El-Tanani, Manfredi Rizzo

**Affiliations:** 1 Department of Anatomy, Faculty of General Medicine, “Ovidius” University, 900470 Constanta, Romania; 2 School of Medicine, PROMISE Department of Health Promotion Sciences Maternal and Infantile Care, Internal Medicine and Medicinal Specialties, University of Palermo, 90133 Palermo, Italy; 3 RAK College of Pharmacy, RAK Medical and Health Sciences University, Ras Al Khaimah 11172, United Arab Emirates; 4 RAK College of Medical Sciences, RAK Medical and Health Sciences University, Ras Al Khaimah 11172, United Arab Emirates; 5 Department of Internal Diseases Propedeutics, Yerevan State Medical University, Yerevan 0025, Armenia; 6 Department and Clinic of Internal Diseases and Metabolic Disorders, Poznań University of Medical Sciences, 61-701 Poznań, Poland; 7 Department of Endocrinology, Diabetes and Metabolism, Elena Venizelou Hospital, GR-11521 Athens, Greece; 8 Department of Pharmacoeconomics and Social Pharmacy, Poznań University of Medical Sciences, 61-701 Poznań, Poland

**Keywords:** mesenteric panniculitis, gut microbiota, gut dysbiosis, intestinal barrier dysfunction, metabolic endotoxemia, glycemic variability, mesenteric adipose tissue, vascular dysfunction, microbiome modulation, precision medicine

## Abstract

Mesenteric panniculitis (MP) is an uncommon inflammatory disorder of mesenteric adipose tissue. Its pathophysiology remains unclear. Gut dysbiosis, intestinal barrier dysfunction, metabolic endotoxemia, glycemic variability (GV), and vascular dysfunction have been implicated in inflammatory and metabolic disorders, but their specific involvement in MP has not been established. This narrative review integrates MP-specific clinical evidence with indirect mechanistic evidence from related metabolic, inflammatory, and experimental settings to examine the possible relationships between these mechanisms and MP and their integration within a proposed gut–mesentery–metabolic axis. The literature was reviewed through structured searches of PubMed, Scopus, and Web of Science for relevant publications from 2018 to 2026, supplemented by earlier foundational studies identified through reference-list screening and targeted searches. Current data suggest that dysbiosis and impaired intestinal barrier function may facilitate microbial-product translocation and lipopolysaccharide-mediated inflammatory signaling, while GV may contribute to oxidative stress, endothelial dysfunction, and pro-inflammatory responses. Mesenteric vascular anatomy and impaired regional perfusion may represent additional factors influencing local tissue susceptibility. Recent randomized controlled trials of microbiome-targeted interventions in metabolic disorders have shown heterogeneous effects on glycemic, inflammatory, and microbiota-related outcomes, indicating a need for further investigation of individualized microbiome-directed strategies. Direct evidence that microbial, metabolic, or vascular mechanisms initiate or sustain MP is currently limited. Accordingly, the proposed gut–mesentery–metabolic axis should be interpreted as a hypothesis-generating framework rather than an established causal model. Prospective MP-specific studies integrating microbiome profiling, validated measures of intestinal barrier function, metabolic phenotyping, GV, vascular assessment, and imaging are required to test the proposed relationships.

## 1. Introduction

Metabolic inflammation is a central feature of obesity [1]. Toll-like receptor 4-mediated innate immune activation contributes to fatty acid-induced insulin resistance [2]. Alterations in the gut microbiota are involved in intestinal and systemic inflammatory processes [3]. Gut-derived low-grade endotoxemia has also been associated with atherothrombosis and cardiovascular disease [4].

Gut dysbiosis and intestinal barrier disruption may contribute to adipose tissue dysfunction in metabolic disorders. Increased intestinal permeability can facilitate the translocation of microbial products into the circulation, thereby promoting systemic inflammatory responses [5].

Mesenteric panniculitis (MP) is a rare inflammatory disorder involving the adipose tissue of the mesentery [6]. Its clinical presentation is heterogeneous and may range from an incidental imaging finding to symptomatic abdominal disease [6]. MP has been described in association with inflammatory, autoimmune, surgical, traumatic, and neoplastic conditions [7]. These associations do not establish a direct causal relationship [7].

Current evidence indicates that gut microbial composition and intestinal barrier integrity are involved in systemic and adipose tissue inflammation [5]. Dysbiosis-associated increases in intestinal permeability may facilitate microbial-product translocation and metabolic endotoxemia [8]. Gut microbiota, intestinal permeability, and systemic inflammation are interconnected [9]. Enteric-derived lipopolysaccharides may contribute to adipose tissue and metabolic inflammation [10].

Glycemic variability (GV) refers to fluctuations in blood glucose concentrations over time [11]. It has been associated with diabetic microvascular and macrovascular complications [11]. Glucose fluctuations may promote oxidative stress, endothelial dysfunction, and cardiovascular injury [12]. GV may exert harmful effects beyond those associated with sustained hyperglycemia alone [12] and may contribute to inflammatory signaling [12,13].

The intestinal vasculature has specialized structural and functional characteristics that contribute to regional tissue homeostasis [14]. These characteristics may also influence local perfusion [14]. Direct evidence that mesenteric vascular variants cause MP is currently lacking.

GV may contribute to vascular dysfunction through oxidative stress and inflammatory signaling [13]. Interactions between the gut microbiota and the host immune system can also promote immune activation and systemic inflammation [15]. The specific contribution of these mechanisms to MP remains insufficiently investigated.

This narrative review examines the potential relationships between gut dysbiosis, intestinal barrier dysfunction, metabolic inflammation, GV, and mesenteric inflammatory susceptibility. We propose the gut–mesentery–metabolic axis as a hypothesis-generating framework that has not yet been established as a pathogenic pathway. Throughout the review, evidence obtained directly from patients with MP is distinguished from indirect human, experimental, and mechanistic evidence derived from other conditions.

This review also considers the potential role of microbiome-targeted strategies in metabolic and inflammatory disorders [16]. Their relevance to MP remains to be established. The recent literature describes the development of microbiome-informed approaches to personalized intervention in metabolic disease [17].

## 2. Materials and Methods

### 2.1. Review Design and Reporting Framework

This article was designed as a structured narrative review examining the potential interactions between mesenteric panniculitis (MP), gut dysbiosis, intestinal barrier dysfunction, microbial translocation, mesenteric vascular anatomy, regional perfusion, glycemic variability (GV), and metabolic inflammation.

This review was developed to integrate anatomical, mechanistic, experimental, imaging, translational, and clinical evidence relevant to the proposed gut–mesentery–metabolic axis. Attention was given to distinguishing established characteristics of MP from biologically plausible mechanisms that have not yet been directly demonstrated in patients with this condition.

The reporting approach was guided by the Scale for the Assessment of Narrative Review Articles (SANRA), with particular attention to the clarity of the review rationale, formulation of the review objective, description of the literature search, appropriate citation of the literature, scientific reasoning, and balanced interpretation of the available evidence [18].

This review was not conducted as a systematic or scoping review. We did not perform a Preferred Reporting Items for Systematic Reviews and Meta-Analyses (PRISMA) flow diagram, formal risk-of-bias assessment, methodological quality assessment, protocol registration, or quantitative synthesis.

### 2.2. Search Strategy

Structured searches were conducted in PubMed, Scopus, and Web of Science for publications from 2018 to 2026, with the last search performed on 18 August 2026. PubMed was used as the primary database for the electronic search strategy (Table S1). Web of Science and Scopus were additionally searched to cross-validate reference retrieval (Supplementary Materials Tables S2 and S3). Of the 77 references included in the final review, 50 were directly retrieved through the PubMed electronic search strategy, while the remaining 27 were identified through citation cross-referencing and targeted retrieval of relevant foundational publications, consistent with SANRA guidance for narrative reviews [18]. Only articles published in English were considered. Earlier publications were also included when they provided foundational clinical, anatomical, histopathological, radiological, or mechanistic information relevant to MP or the conceptual framework of the review. These earlier studies were identified through reference-list screening and targeted retrieval of key publications. Google Scholar was used only as a supplementary source to locate individual articles identified through reference lists or prior knowledge and was not included as a separate structured search strategy.

The search combined terms related to MP and mesenteric inflammation, including “mesenteric panniculitis”, “sclerosing mesenteritis”, “retractile mesenteritis”, “mesenteric lipodystrophy”, “misty mesentery”, “mesenteric inflammation”, and “mesenteric adipose tissue”.

Terms related to the gut microbiota and intestinal barrier included “gut microbiota”, “gut microbiome”, “dysbiosis”, “intestinal permeability”, “intestinal barrier dysfunction”, “leaky gut”, “microbial translocation”, “lipopolysaccharide”, “LPS”, “metabolic endotoxemia”, “short-chain fatty acids”, “butyrate”, “Toll-like receptor 4”, “TLR4”, and “NF-κB”, “microbiome modulation”, “microbiota-targeted therapy”, “microbiome-targeted intervention”, “precision microbiome”, “probiotic”, “prebiotic”, “synbiotic”, “postbiotic”, “personalized nutrition”, and “randomized controlled trial”.

Terms related to vascular anatomy and perfusion included “mesenteric vascular anatomy”, “mesenteric arterial variants”, “superior mesenteric artery”, “inferior mesenteric artery”, “celiac trunk”, “mesenteric perfusion”, “mesenteric ischemia”, “chronic mesenteric ischemia”, “ischemia–reperfusion injury”, “endothelial dysfunction”, “microvascular dysfunction”, and “hypoxia-inducible factor 1-alpha”.

Terms related to metabolic dysfunction included “glycemic variability”, “glucose variability”, “glucose fluctuations”, “continuous glucose monitoring”, “oxidative stress”, “metabolic inflammation”, “type 2 diabetes”, “insulin resistance”, “visceral adiposity”, “obesity”, and “metabolic syndrome”.

The reference lists of selected publications were also screened to identify additional relevant studies not retrieved through the initial database search. The literature search used a two-level strategy. First, disease-specific searches were performed using terms related to MP and sclerosing mesenteritis to identify direct clinical, radiological, pathological, and epidemiological evidence. Second, broader thematic searches were performed separately for gut microbiota and intestinal barrier dysfunction, metabolic endotoxemia, GV, and vascular mechanisms. This approach was chosen because direct studies simultaneously addressing MP and these mechanistic domains were scarce or absent. Cross-domain searches combining MP-related terms with the principal mechanistic concepts were also performed to assess the availability of direct MP-specific evidence. When these combined searches retrieved no or very limited MP-specific evidence, the corresponding thematic literature was used only as indirect mechanistic evidence and was explicitly distinguished from evidence obtained directly in patients with MP.

Full database-specific search strategies and record counts are provided in Supplementary Materials Tables S1–S3.

### 2.3. Selection Criteria

We considered human, animal, experimental, translational, anatomical, imaging, clinical, and observational studies, as well as systematic reviews, meta-analyses, and narrative reviews when they addressed MP or mechanisms potentially relevant to mesenteric inflammation.

Studies were considered eligible when they addressed at least one of the principal themes of the review: clinical manifestations of MP, radiological characteristics, histopathological findings, mesenteric adipose tissue inflammation, gut dysbiosis, intestinal barrier dysfunction, microbial translocation, metabolic endotoxemia, mesenteric vascular anatomy, vascular anatomical variants, regional perfusion abnormalities, chronic low-grade ischemia, GV, oxidative stress, endothelial dysfunction, insulin resistance, or metabolic inflammation.

Studies investigating gastrointestinal pathogens, including *Helicobacter pylori*, were considered when they provided mechanistic evidence regarding dysbiosis, intestinal barrier disruption, microbial-product dissemination, systemic inflammation, or metabolic dysfunction. Because direct evidence connecting individual gastrointestinal pathogens to MP is limited, these findings were interpreted as indirect mechanistic evidence.

Studies examining probiotics, prebiotics, postbiotics, short-chain fatty acids (SCFAs), glucose-lowering therapies, vascular interventions, anti-inflammatory therapies, or other microbiome-targeted approaches were considered when they addressed mechanisms potentially relevant to the proposed gut–mesentery–metabolic axis.

Randomized controlled trials of microbiome-targeted interventions were considered when they evaluated microbial, metabolic, inflammatory, intestinal barrier, or cardiometabolic outcomes relevant to the proposed gut–mesentery–metabolic axis.

We excluded conference abstracts without accessible full text, as well as letters, comments, and editorials without relevant mechanistic or clinical discussion. We also excluded isolated case reports that did not provide relevant diagnostic, therapeutic, pathological, or mechanistic information.

Non-English publications were excluded. Studies without a clear connection to MP, mesenteric inflammation, gut barrier dysfunction, dysbiosis, vascular perfusion, GV, or metabolic inflammation were also excluded.

The inclusion and exclusion criteria are summarized in Supplementary Materials Table S4.

### 2.4. Study Selection and Author Involvement

The literature search and initial organization of the retrieved material were coordinated by S.I., V.M., M.E.-T., and A.F.W., with overall scientific supervision from M.R. Titles and abstracts were screened for relevance by S.I., V.M., A.F.W., S.T., S.A.R., B.A.B., I.R.R., and S.M.S. according to the predefined thematic areas of the review.

Retrieved records were manually screened by title and abstract for thematic relevance. Potentially relevant articles were then assessed in full text. Studies were retained when they provided direct clinical, imaging, pathological, or epidemiological evidence on MP or indirect mechanistic evidence relevant to the predefined domains of gut microbiota, intestinal barrier dysfunction, metabolic endotoxemia, GV, or vascular biology. Duplicate records and clearly irrelevant articles were excluded. Because this was a narrative review, no formal PRISMA-based screening process or risk-of-bias assessment was performed.

The anatomical and vascular literature was reviewed with particular attention to mesenteric continuity, arterial anatomy, vascular variants, collateral circulation, and regional perfusion. The clinical and imaging literature was evaluated for evidence concerning the diagnosis, differential diagnosis, natural history, and management of MP.

The microbiota-related literature was assessed for evidence regarding dysbiosis, intestinal barrier dysfunction, microbial translocation, lipopolysaccharide-mediated signaling, and metabolic endotoxemia. The metabolic literature was evaluated for evidence regarding GV, oxidative stress, endothelial dysfunction, adipose tissue inflammation, insulin resistance, and glucose-lowering therapies.

A.A., K.H., I.I., and A.P. contributed to the clinical, cardiovascular, endocrine, metabolic, and pharmacoeconomic interpretation of the evidence. Uncertainties regarding article relevance, thematic classification, or interpretation were resolved through discussion between the authors, with final oversight by M.E.-T. and M.R.

### 2.5. Evidence Synthesis

The selected literature was synthesized narratively and organized into thematic sections. The synthesis focused on the clinical, pathological, and imaging characteristics of MP; gut dysbiosis and intestinal barrier dysfunction; microbial translocation and lipopolysaccharide-mediated immune activation; the potential systemic effects of gastrointestinal pathogens; mesenteric vascular anatomy and perfusion; GV; microbiome-targeted interventions and evidence from randomized controlled trials; and the possible relationships between these domains within a gut–mesentery–metabolic axis.

Because the included evidence originated from heterogeneous study designs, mechanistic plausibility was distinguished from direct clinical evidence whenever possible. Findings derived from animal models, cellular studies, anatomical investigations, and indirect metabolic evidence were interpreted separately from observations obtained directly from patients with MP.

Attention was given to avoiding causal interpretations when the available evidence demonstrated only biological plausibility or epidemiological association. Established diagnostic and pathological features of MP were separated from proposed mechanisms involving dysbiosis, vascular susceptibility, chronic low-grade ischemia, and GV.

The strength of the evidence was interpreted according to its origin. Direct clinical, imaging, or histopathological evidence in patients with MP was considered separately from evidence derived from patients with obesity, type 2 diabetes, metabolic syndrome, inflammatory bowel disease, or other disorders involving visceral adipose inflammation.

Selected mechanisms, imaging findings, and representative studies were summarized in tables to improve clarity and facilitate clinical interpretation. The proposed gut–mesentery–metabolic axis was used as an integrative, hypothesis-generating framework rather than as an established causal model.

## 3. Mesenteric Panniculitis: Clinical and Pathological Overview

### 3.1. Epidemiology

Mesenteric panniculitis (MP) is an uncommon chronic inflammatory disorder involving the adipose tissue of the mesentery [19]. The mesentery has recently been redefined in anatomical research as a continuous organ with distinct functional characteristics [20]. The condition is frequently identified incidentally during abdominal computed tomography (CT) performed for unrelated clinical indications [21].

The true prevalence of MP remains uncertain because diagnostic criteria, imaging protocols, study populations, and radiological reporting practices differ considerably between published studies [21]. In a CT study conducted by Daskalogiannaki et al., MP was identified in approximately 0.6% of patients examined [22]. More recent reviews have reported prevalence estimates ranging from approximately 0.16% to 7.8% among individuals undergoing abdominal CT [21].

A substantial proportion of patients may remain asymptomatic. When symptoms are present, abdominal pain is the most frequently reported manifestation. Other possible manifestations include abdominal distension, bloating, altered bowel habits, nausea, vomiting, anorexia, fever, fatigue, and weight loss [21].

MP is diagnosed more frequently in men than in women [23]. Published clinical series have reported male-to-female ratios ranging from approximately 2:1 to 3:1 [23]. The disorder predominantly affects middle-aged and older adults, with most patients diagnosed in the sixth and seventh decades of life [19]. Nevertheless, cases have also been described outside this age range [24].

Several clinical conditions have been reported in association with MP. Previous abdominal surgery or trauma has been documented in a proportion of affected patients [25]. Autoimmune and inflammatory disorders have also been associated with the condition [7]. Metabolic syndrome and diabetes mellitus have been reported among patients with MP [24]. Vascular disorders have also been reported more frequently in some MP cohorts [24]. Recent disease-specific evidence provides additional support for an association between MP and cardiometabolic comorbidity. In a matched case–control study, Schweistein et al. reported higher frequencies of dyslipidemia, hypertension, obesity, and non-alcoholic fatty liver disease among patients with MP than among controls, with dyslipidemia, obesity, and fatty liver disease remaining independently associated with MP in multivariable analysis [26]. These findings support an epidemiological association between MP and selected cardiometabolic risk factors; however, they do not establish causality.

An association between MP and malignancy has been reported in observational and imaging studies, although its strength and clinical significance remain controversial. Selection and imaging-related biases may partly explain the apparent association because patients with malignancy undergo abdominal imaging more frequently than the general population [24]. MP should not be interpreted as a paraneoplastic condition based solely on its radiological identification [24].

The available epidemiological evidence does not establish a specific causal relationship between MP and metabolic, autoimmune, surgical, traumatic, or neoplastic conditions [7]. MP may instead represent a heterogeneous radiological and pathological response to different local or systemic inflammatory stimuli [19].

Recent cohort studies further emphasize the clinical heterogeneity of MP and sclerosing mesenteritis. In a large single-center series of patients with sclerosing mesenteritis, abdominal symptoms were common, although only a subset were considered directly attributable to the mesenteric process [27]. Similarly, a recent cohort of patients with radiologically diagnosed MP showed that 31.5% were asymptomatic and 85.6% remained clinically stable during follow-up, indicating a generally indolent course in primary forms [28]. These observations reinforce the need to distinguish incidental radiological abnormalities from clinically significant disease.

### 3.2. Histopathology

Mesenteric lipodystrophy, MP, and retractile mesenteritis have historically been described as overlapping histopathological patterns within the spectrum of sclerosing mesenteritis. These patterns reflect the varying predominances of fat necrosis, chronic inflammation, and fibrosis and should not be interpreted as obligatory sequential stages of disease progression. Histological specimens may demonstrate mixed features, with variable contributions from inflammation, fat necrosis, and fibrosis [29].

Chronic inflammatory infiltrates composed of plasma cells, histiocytes, and lymphocytes, sometimes with a perivascular distribution, have also been described [30].

Although histopathological confirmation is the most definitive diagnostic method, it is rarely obtained unless malignancy is suspected because diagnosis primarily relies on radiological findings [21,24].

A crucial consideration during tissue evaluation is the differential diagnosis of lymphoma and metastases [24] and Immunoglobulin G4 (IgG4)-related disease [31]. In some cases, fibroblastic proliferation may have features similar to those of desmoid-type fibromatosis without the defining features of true neoplastic fibromatosis [24,32]. Desmoid-type fibromatosis is a rare, locally aggressive fibroblastic neoplasm that does not metastasize but may infiltrate adjacent structures and recur after treatment [33].

### 3.3. Imaging

The diagnosis of MP depends primarily on imaging, particularly computed tomography (CT). Features such as well-defined areas of increased fat attenuation, the “fat ring sign,” and a pseudocapsule are important diagnostic findings [19]. The fat ring sign, i.e., preservation of normal fat density around mesenteric vessels and nodes, helps differentiate MP from neoplastic or infectious processes [22].

Contemporary expert guidance emphasizes that the diagnosis of sclerosing mesenteritis should integrate characteristic cross-sectional imaging findings with the clinical context and careful exclusion of relevant mimics [34]. The 2025 American Gastroenterological Association (AGA) Clinical Practice Update provides current recommendations for the diagnostic evaluation and management of sclerosing mesenteritis [34], while a recent Mayo Clinic clinical review highlights the central role of CT in contemporary diagnostic practice [35]. Histopathological confirmation is not routinely required when imaging findings are characteristic. Biopsy may be warranted when the diagnosis remains uncertain or malignancy cannot be confidently excluded [35].

Although less commonly used than CT, magnetic resonance imaging (MRI) serves as an alternative diagnostic tool, with signal characteristics varying according to the relative proportions of inflammation and fibrosis [19].

Positron emission tomography combined with CT (PET/CT) may contribute to the differential diagnosis of MP and malignancy when mesenteric lesions show absent fluorodeoxyglucose uptake [19].

Occasionally, increased echogenicity of the mesenteric fat and hypoechoic nodules or bands can be detected by ultrasound [24]. Ultrasound has limited diagnostic value for deep mesenteric abnormalities compared with CT [19].

Imaging facilitates diagnosis and may contribute to the assessment of disease evolution. Imaging findings must be interpreted in the clinical context because similar patterns may occur in lymphoma, peritoneal carcinomatosis, or desmoid tumors [24].

Mesenteric neoplasms, including lymphoma, metastatic carcinoid tumor, desmoid tumor, and mesenteric carcinomatosis, may mimic sclerosing mesenteritis on imaging; when clinical suspicion remains equivocal, biopsy may be warranted for definitive diagnosis [35].

### 3.4. Current Management and Unmet Therapeutic Needs

Current expert guidance supports a symptom-oriented approach to the management of sclerosing mesenteritis. Asymptomatic patients are generally observed without treatment, whereas medical therapy is reserved for patients with clinically significant symptoms or complications [34]. The primary goal of treatment is symptom relief rather than radiological normalization, as imaging abnormalities may persist despite clinical improvement [35]. For symptomatic disease, corticosteroid-based regimens, often combined with tamoxifen, have been used in clinical practice; however, the supporting evidence is derived predominantly from observational studies and retrospective series rather than randomized controlled trials [35].

Immunomodulatory agents, including azathioprine and methotrexate, have been used in patients with persistent or refractory symptoms [19]. Surgical intervention is generally reserved for severe complications, including recurrent or persistent intestinal obstruction [21]. Surgery is not considered a routine curative treatment for MP [19].

Associated disorders should be identified and managed according to their specific clinical indications [21]. The therapeutic approach to MP should be individualized, balancing symptom severity, comorbidities, and potential treatment risks [23]. Further prospective studies are necessary to validate treatment algorithms and identify prognostic factors that may guide long-term management.

## 4. Gut Dysbiosis, Intestinal Barrier Dysfunction, and Mesenteric Inflammation

### 4.1. Definition and Composition of Dysbiosis

The gut microbiota is a complex microbial ecosystem composed predominantly of bacteria, together with archaea, fungi, protozoa, and viruses [36]. In healthy individuals, the bacterial component is characterized by substantial diversity and is dominated mainly by the phyla *Firmicutes*, *Bacteroidetes*, *Actinobacteria*, and *Proteobacteria* [3].

Dysbiosis refers to an alteration in the composition, diversity, distribution, or functional activity of the microbiota that disrupts the homeostatic relationship between the host and its microorganisms [37].

Table 1 highlights the transition from a balanced microbial ecosystem, characterized by homeostatic and protective functions, to a dysbiotic ecosystem marked by the loss of beneficial microorganisms, the expansion of pathobionts, reduced microbial diversity, and altered metabolic activity [37]. Dysbiosis may impair intestinal barrier integrity and increase intestinal permeability [38]. These changes can activate inflammatory pathways and contribute to systemic metabolic dysfunction [39]. Interactions between gut dysbiosis and mesenteric adipose tissue inflammation have been described in inflammatory bowel disease, although direct evidence in mesenteric panniculitis (MP) remains limited [40].

Dysbiosis commonly involves the loss of beneficial microorganisms; expansion of pathobionts, including *Enterobacteriaceae*; and reduced overall microbial diversity [37].

Western-style diets rich in fat and low in fiber may promote gut dysbiosis, intestinal barrier dysfunction, and metabolic inflammation [41]. Antibiotic exposure may also alter the composition and functional activity of the gut microbiota [37]. Obesity and type 2 diabetes mellitus are frequently associated with dysbiotic microbial profiles [39].

Dysbiosis alters the production and availability of microbial metabolites, including short-chain fatty acids (SCFAs), bile acid derivatives, lipopolysaccharides, and branched-chain amino acids [39]. Microbiota-derived metabolites influence host metabolic homeostasis and immune function [42]. Reduced production of beneficial metabolites such as butyrate may impair epithelial barrier integrity and anti-inflammatory regulation [42].

Intestinal barrier dysfunction associated with dysbiosis facilitates the translocation of lipopolysaccharides into the circulation [4]. Lipopolysaccharides activate Toll-like receptor 4 signaling and promote inflammatory and metabolic responses [2]. Toll-like receptor 4 activation promotes nuclear factor kappa B-dependent inflammatory signaling [2]. This signaling increases the production of pro-inflammatory mediators, including tumor necrosis factor alpha, interleukin-6, and interleukin-1β [4].

Dysbiosis is involved in the pathophysiology of inflammatory bowel disease [44]. Dysbiotic microbial profiles have also been associated with obesity and type 2 diabetes mellitus [39]. Diet-induced dysbiosis and intestinal inflammation contribute to the development of metabolic dysfunction-associated fatty liver disease [41].

Intestinal barrier disruption and microbial translocation may promote inflammatory signaling in tissues adjacent to the gut [38]. E-cadherin dysfunction may further impair epithelial barrier integrity and facilitate bacterial invasion or dissemination [45]. Interactions between the gut microbiota and mesenteric adipose tissue have been described in inflammatory intestinal disease [40]. Direct evidence linking these mechanisms to MP remains insufficient. Mesenteric adipose tissue inflammation described in Crohn’s disease, obesity, or other metabolic disorders should not be considered equivalent to MP. Evidence from these conditions is used here only as indirect mechanistic support.

The mesentery is an anatomically continuous and functionally active structure containing vascular, lymphatic, adipose, and immune components [20]. Evidence of interactions between the gut microbiota and mesenteric adipose tissue supports the proposed concept of a gut–mesentery axis [40]. This concept may have implications for gastrointestinal, inflammatory, and metabolic disorders.

### 4.2. Intestinal Barrier Dysfunction and Microbial Translocation

An important mechanistic link between gut dysbiosis and mesenteric and systemic inflammation is increased intestinal permeability [47]. Increased intestinal permeability facilitates the translocation of microbial products, including lipopolysaccharides, into the circulation [5]. Dysbiosis and microbial-product translocation may contribute to systemic metabolic inflammation [39]. Metabolic endotoxemia induced by circulating lipopolysaccharides may further amplify systemic inflammatory responses [8].

In a healthy intestinal barrier, epithelial cells and the protective mucus layer act synergistically to prevent the passage of microbial antigens into subepithelial tissues [43]. Tight junction proteins, including cingulin, tricellulin, occludin, claudins, zonula occludens-1, and junctional adhesion molecules, regulate paracellular permeability [43]. *Akkermansia muciniphila*-derived extracellular vesicles may influence intestinal permeability through the regulation of tight junction proteins [48]. Gut barrier dysfunction is also associated with adipose tissue inflammation and metabolic disorders [5].

In the context of dysbiosis, dietary imbalances, or metabolic stress, the intestinal barrier may become compromised [49]. This increase in intestinal permeability is commonly described as “leaky gut” [49].

Under physiological conditions, a balanced gut microbiota contributes to epithelial integrity, regulates the production of SCFAs, and supports immune tolerance [3]. A healthy intestinal barrier also limits the passage of microbial antigens and products into the underlying tissues and circulation [43].

When dysbiosis occurs, beneficial microbial populations may decline and pathobionts may expand [37]. Dysbiosis may also reduce the production of protective microbial metabolites such as butyrate [50]. Reduced butyrate availability may impair tight-junction integrity and weaken intestinal barrier function [50].

Intestinal barrier disruption facilitates the translocation of lipopolysaccharides and other microbial products into the circulation [9]. Lipopolysaccharides activate Toll-like receptor 4 signaling in macrophages and adipocytes [2]. Toll-like receptor 4 activation promotes nuclear factor kappa B-dependent inflammatory signaling [2]. This signaling increases the production of pro-inflammatory mediators, including tumor necrosis factor alpha, interleukin-6, and interleukin-1β [4].

Enteric-derived lipopolysaccharides may promote macrophage activation, adipocyte dysfunction, and chronic low-grade inflammation [10]. Direct evidence that lipopolysaccharide exposure causes early fibrosis, specifically in MP, remains lacking.

Chronic dysbiosis-associated inflammation may affect mesenteric adipose tissue in inflammatory intestinal disorders [46]. Interactions between the gut microbiota, intestinal inflammation, and mesenteric adipose tissue have been described in Crohn’s disease [40]. These findings provide indirect mechanistic support for mesenteric immune activation but do not establish the same pathway in MP.

Microbial-product translocation contributes to metabolic endotoxemia and systemic inflammation [8]. Metabolic endotoxemia may promote insulin resistance through inflammatory and innate immune pathways [8]. Diet-induced dysbiosis and intestinal inflammation may also contribute to metabolic dysfunction-associated steatotic liver disease [41].

Lipopolysaccharide is a major structural component of the outer membrane of Gram-negative bacteria and a potent microbe-associated molecular pattern [51]. Persistent exposure to circulating lipopolysaccharides may promote systemic and adipose tissue inflammation [4]. Enteric-derived lipopolysaccharides have also been implicated in obesity-associated metabolic dysfunction [10]. Their specific causal contribution to MP has not been established.

Experimental and translational evidence indicates that metabolic endotoxemia contributes to systemic inflammation and insulin resistance [8]. Dietary factors may alter gut microbiota composition, increase intestinal permeability, and elevate circulating lipopolysaccharide concentrations [52]. In high-fat-diet-fed mice, selected lactic acid bacterial strains may improve microbial composition and modulate metabolic and immunological parameters [53]. These experimental findings support microbiota-targeted mechanisms but cannot be directly extrapolated to the treatment of MP.

The mesentery is a continuous anatomical structure containing adipose tissue, vessels, lymphatics, connective tissue, and immune components [54]. Its anatomical relationship with the intestine allows it to participate in immune surveillance, lymphatic drainage, and inflammatory responses [54]. In inflammatory bowel disease, mesenteric adipose tissue may become an active source of adipokines and inflammatory mediators [46]. Crosstalk between the intestinal microbiota and mesenteric adipose tissue has also been described in Crohn’s disease [40].

MP is histopathologically characterized by varying proportions of fat necrosis, chronic inflammation, and fibrosis [23]. These pathological characteristics should not be attributed directly to lipopolysaccharide exposure without disease-specific experimental or clinical evidence.

Elevated circulating lipopolysaccharide and zonulin concentrations have been reported in patients with type 2 diabetes mellitus [55]. Elevated plasma zonulin concentrations have also been described in obesity-associated fatty liver disease [56]. These biomarkers require cautious interpretation. Commercial assays commonly used to measure circulating zonulin may detect proteins other than pre-haptoglobin-2, raising concerns regarding their specificity as direct measures of intestinal permeability [57]. Circulating Lipopolysaccharide (LPS) measurements should also be interpreted cautiously because commonly used assays, including the Limulus amebocyte lysate assay, are affected by methodological and biological factors that may influence LPS detection and quantification [8]. Circulating LPS concentrations should not be considered a direct or definitive measure of biologically active endotoxemia.

These findings support an association between intestinal barrier dysfunction and metabolic disease, but they do not demonstrate a correlation between zonulin and radiological signs of MP.

Increased intestinal permeability and microbial-product translocation may contribute to systemic metabolic inflammation [9]. These mechanisms may also influence immune responses in tissues adjacent to the intestine [40]. Their role in MP remains biologically plausible but unproven. The proposed gut–barrier–mesentery pathway should be considered a framework for future investigation.

*Helicobacter pylori* infection has been associated with alterations in gastric and gut microbiota composition [58] and has been investigated in relation to metabolic syndrome [59]. It has also been studied as a potential contributor to extra-gastric vascular injury, particularly endothelial dysfunction, although its cardiovascular relevance remains uncertain and context-dependent [60]. No direct evidence currently links *H. pylori* infection or these vascular effects to MP.

### 4.3. Metabolic Endotoxemia and LPS-Mediated Inflammatory Signaling

Once lipopolysaccharide reaches adipose tissue, it may be recognized by Toll-like receptor 4 expressed on macrophages and adipocytes. Toll-like receptor 4 activation initiates intracellular inflammatory signaling, including nuclear factor kappa B-dependent pathways. This signaling promotes the release of pro-inflammatory mediators and contributes to insulin resistance [2]. Gut-derived low-grade endotoxemia is also associated with systemic inflammatory and vascular responses [4].

Dysbiosis may increase the relative abundance of Gram-negative bacteria and alter the production and availability of bacterial lipopolysaccharides [9]. Intestinal barrier dysfunction facilitates the translocation of lipopolysaccharides from the intestinal lumen into the circulation [49]. Gut microbiota alterations, intestinal permeability, and systemic inflammation are interconnected [9]. Circulating lipopolysaccharides activate Toll-like receptor 4-dependent inflammatory pathways in macrophages and adipocytes [2]. Toll-like receptor 4 signaling promotes nuclear factor kappa B activation and contributes to chronic low-grade inflammation [2]. Persistent low-grade endotoxemia has been associated with systemic inflammation, endothelial dysfunction, and atherothrombotic risk [4].

The mesentery may be a target of gut-derived inflammatory signals because it contains adipose tissue, immune cells, blood vessels, and lymphatic structures [54]. Interactions between intestinal inflammation and mesenteric adipose tissue have been described in inflammatory bowel disease [46]. Crosstalk between the gut microbiota and mesenteric adipose tissue has also been reported in Crohn’s disease [40]. Direct evidence that lipopolysaccharide exposure specifically causes MP remains lacking.

Lipopolysaccharides may induce adipocyte dysfunction and promote obesity-associated metabolic inflammation [10]. Metabolic endotoxemia contributes to systemic inflammatory responses and insulin resistance [8]. Toll-like receptor 4 activation also interferes with insulin signaling and contributes to fatty acid-induced insulin resistance [2].

Diet-induced dysbiosis and intestinal inflammation may contribute to hepatic steatosis and metabolic dysfunction-associated steatotic liver disease [41]. Gut microbiota alterations, intestinal permeability, and circulating microbial products participate in a metabolic–inflammatory network involving the intestine, adipose tissue, liver, and vascular system [9].

MP is characterized histologically by varying degrees of fat necrosis, chronic inflammation, and fibrosis [23]. These pathological changes should not be attributed directly to lipopolysaccharide exposure because disease-specific experimental evidence is currently insufficient.

### 4.4. Microbiome-Targeted Interventions: Current Evidence and Translational Potential

The mesentery is a continuous anatomical and functional structure containing adipose tissue, immune cells, blood vessels, and lymphatic components [54]. Gut-derived low-grade endotoxemia may promote systemic inflammatory and vascular responses [4].

Elevated circulating lipopolysaccharide concentrations have been reported in patients with type 2 diabetes mellitus [55]. Elevated plasma zonulin concentrations have also been observed in obesity-associated fatty liver disease [56]. These studies did not demonstrate a direct correlation between circulating lipopolysaccharide or zonulin concentrations and radiological signs of MP.

#### 4.4.1. Probiotics, Prebiotics, Synbiotics, and Postbiotics

Probiotics and prebiotics may contribute to the restoration of microbial balance and the modulation of metabolic endotoxemia [52]. Selected lactic acid bacterial strains have improved microbial, metabolic, and immunological parameters in high-fat-diet-fed mice [53].

Some randomized controlled trials have investigated microbiome-targeted interventions in patients with metabolic disorders. In a randomized, double-blind, placebo-controlled trial involving 120 patients with type 2 diabetes, a synbiotic containing *Bifidobacterium animalis* subsp. *lactis* and *galactooligosaccharide* reduced fasting blood glucose and modified inflammatory markers, oxidative stress, gut microbiota composition, bile acids, and Glucagon-like peptide-1 (GLP-1) concentrations [61]. The synbiotic intervention produced greater effects on fasting glucose than the probiotic alone, although between-group differences were not demonstrated for all glycemic outcomes [61].

#### 4.4.2. Short-Chain Fatty Acids and Barrier-Directed Strategies

Butyrate may reinforce intestinal barrier function and reduce inflammatory responses in experimental models [62]. Butyrate has also attenuated lipopolysaccharide-induced intestinal inflammation and epithelial injury in animal studies [63]. These findings support microbiome-targeted mechanisms, but direct therapeutic evidence in MP remains lacking.

SCFAs represent an important functional link between microbiota composition, intestinal barrier integrity, immune regulation, and metabolic homeostasis. The clinical effects of interventions designed to increase SCFA production depend on baseline microbiota composition, diet, metabolic phenotype, and the specific intervention used [17].

#### 4.4.3. Evidence from Recent Randomized Controlled Trials

A randomized, placebo-controlled study evaluating the synbiotic formulation EDC-HHA01 in adults with prediabetes or type 2 diabetes reported stage-dependent effects, with significant improvements in glycated hemoglobin A1c (HbA1c), fasting insulin, insulin resistance indices, and markers of metabolic endotoxemia in participants with prediabetes. Effects were more modest and did not reach statistical significance in participants with established type 2 diabetes [64].

The metabolic response to microbiome-targeted interventions may vary according to disease stage and baseline metabolic status. This heterogeneity is relevant to precision microbiome approaches, in which patient stratification may be required before intervention selection.

Recent randomized controlled trials of microbiome-targeted interventions are summarized in Table 2.

These trials were conducted in populations with metabolic disorders and are presented here only as indirect evidence. No randomized controlled trial has evaluated microbiome-targeted therapy in patients with MP.

The heterogeneous responses observed across recent randomized controlled trials suggest that the effects of microbiome-targeted interventions may depend on baseline metabolic status, disease stage, intervention composition, and individual microbiome characteristics.

## 5. Vascular Anatomy Variants and Mesenteric Ischemia

### 5.1. Normal Anatomy and Variants of the Mesenteric Arteries

The gastrointestinal tract and mesenteric adipose tissue receive their arterial supply through the mesenteric vascular system [67]. The principal unpaired anterior branches of the abdominal aorta supplying the gastrointestinal tract are the celiac trunk, superior mesenteric artery, and inferior mesenteric artery. Collateral arcades and arterial anastomoses connect these vascular territories and help maintain intestinal perfusion [68]. The mesentery is currently understood as a continuous anatomical structure containing arterial, venous, lymphatic, neural, adipose, and connective tissue components [20]. Anatomical variants of the mesenteric arterial system are clinically relevant to abdominal surgery and vascular imaging; however, no direct evidence currently links these variants to mesenteric panniculitis (MP).

### 5.2. Potential Relationship Between Vascular Dysfunction and Mesenteric Inflammation

Visceral adipose tissue is highly vascularized and metabolically active [69]. In obesity and cardiometabolic disease, microvascular dysfunction and tissue hypoxia have been associated with adipose tissue inflammation and insulin resistance [70,71]. Chronic mesenteric ischemia is typically related to significant arterial obstruction, and ischemia–reperfusion may promote oxidative and inflammatory signaling [72,73]. These observations provide indirect biological plausibility for a relationship between impaired perfusion and adipose tissue inflammation but do not establish an ischemic mechanism in MP. Direct evidence linking mesenteric vascular variants, chronic low-grade ischemia, or microvascular dysfunction to MP remains lacking.

### 5.3. Imaging and Pathological Evidence

Contrast-enhanced computed tomography (CT) remains the principal imaging modality for evaluating MP, with typical findings including increased attenuation of mesenteric fat, soft-tissue nodules, a fat ring sign, and a pseudocapsule [74]. Vascular encasement may occur within inflamed mesenteric tissue, but this finding should not be interpreted as evidence of arterial stenosis or ischemia [74]. Direct evidence that chronic mesenteric ischemia initiates or perpetuates MP is currently lacking.

## 6. Glycemic Variability and Inflammatory Responses

### 6.1. Definition and Clinical Impact

Glycemic variability (GV) refers to fluctuations in blood glucose concentrations over time and includes short-term intraday and long-term interday variations. It reflects dynamic changes in glucose homeostasis, including episodes of hyperglycemia and hypoglycemia. Huang L. et al. (2023) analyzed short-term GV using continuous glucose monitoring (CGM) metrics, including mean amplitude of glycemic excursions (MAGE) [11]. Long-term GV can be assessed through variability in serial HbA1c or fasting glucose measurements [11].

Clinical evidence identifies GV as a risk marker associated with microvascular and macrovascular complications, including retinopathy, nephropathy, and cardiovascular events [11].

Interactions between gut microbiota composition and glucose metabolism have also been described, with short-chain fatty acids (SCFAs) representing one potential mechanistic pathway [75]. Whether GV contributes to inflammatory remodeling of mesenteric adipose tissue is unknown. Evidence from obesity and insulin resistance indicates that hypoxia and inflammatory remodeling can occur in visceral adipose tissue [71], but these findings cannot be directly extrapolated to mesenteric panniculitis (MP).

Clinically, higher GV is associated with adverse diabetes-related outcomes [11]. Its specific relevance to MP remains unknown.

### 6.2. Role in Modulating Inflammation

The pathophysiological impact of GV is primarily driven by its ability to trigger oxidative stress, endothelial dysfunction, and pro-inflammatory signaling. Oscillating glucose levels generate higher levels of reactive oxygen species (ROS) than sustained hyperglycemia, thereby amplifying oxidative damage in vascular and immune cells [12].

Central transcription factors, including Nuclear factor kappa B (NF-κB), are activated by this oxidative environment, which, in turn, enhances the expression of pro-inflammatory cytokines, including IL-6, IL-1β, and TNF-α [76].

In visceral adipose tissue, metabolic dysfunction is associated with adipocyte hypertrophy, immune cell infiltration, and a shift toward a pro-inflammatory phenotype [70]. Direct evidence that GV specifically induces these changes in mesenteric adipose tissue or MP remains limited.

Glucose fluctuations may contribute to cellular stress through mitochondrial and endoplasmic reticulum-related mechanisms [76].

GV has been associated with oxidative stress, pro-inflammatory signaling, and endothelial dysfunction in diabetes and other metabolic settings [13]. Adipose tissue microcirculatory dysfunction may contribute to inflammatory responses in metabolically dysfunctional adipose tissue [70]. Tissue hypoxia may further promote adipose tissue inflammation and insulin resistance [71]. However, whether these mechanisms contribute to mesenteric inflammatory susceptibility or MP remains unknown.

### 6.3. Links with Dysbiosis and Mesenteric Inflammation

New perspectives on how metabolic dysfunction may contribute to gastrointestinal and immunological disturbances have emerged from recent integrative research examining the interaction between GV and gut dysbiosis [75]. The relationship between these processes and mesenteric inflammation remains largely hypothetical.

SCFAs, particularly butyrate, are important microbial metabolites involved in maintaining intestinal barrier integrity and regulating inflammatory responses [75].

Intestinal barrier dysfunction facilitates the translocation of microbe-associated molecular patterns, including lipopolysaccharides, into the circulation [8]. Lipopolysaccharides activate Toll-like receptor 4 signaling, which promotes pro-inflammatory signaling and contributes to insulin resistance [2]. Direct evidence that these processes occur specifically in mesenteric immune cells in MP remains insufficient.

GV may potentiate oxidative stress and vascular dysfunction [13]. These effects may increase tissue susceptibility to inflammatory injury, although direct evidence that GV induces a specific pro-inflammatory mesenteric microenvironment is currently lacking.

Bidirectional interactions exist between glucose metabolism and the gut microbiota [75]. Microbial metabolites and endotoxins can impair insulin signaling and glucose metabolism [8]. Toll-like receptor 4 activation contributes to insulin resistance [2].

These interactions may create a vicious cycle of dysglycemia and inflammation. Increased circulating lipopolysaccharide concentrations may contribute to insulin resistance through Toll-like receptor 4 (TLR4)-mediated pathways [2]. The extension of this mechanism to persistent mesenteric immune activation remains biologically plausible but unproven.

Current evidence does not establish a direct association between elevated GV, zonulin concentrations, and imaging features of MP.

**Figure 1:**
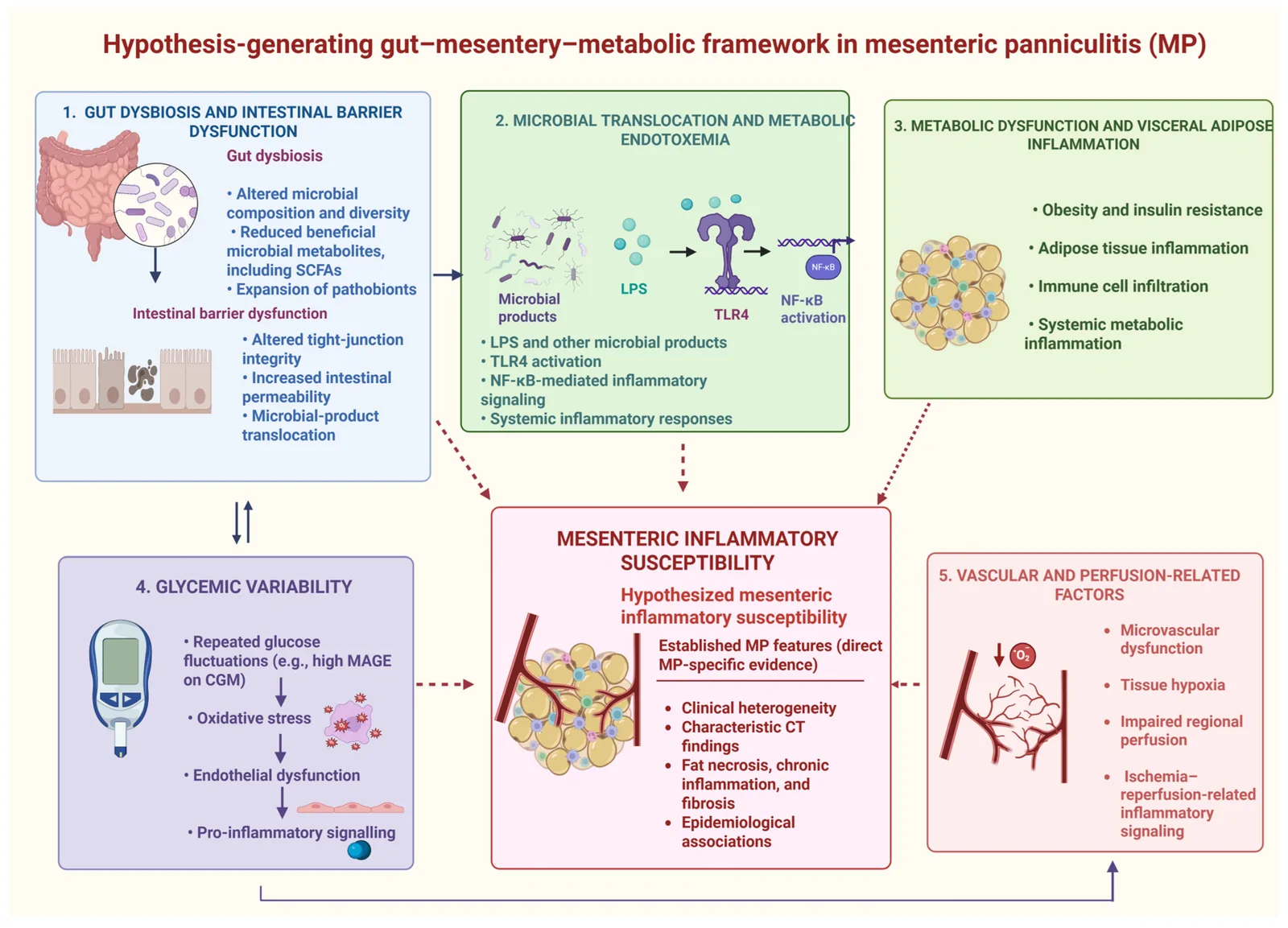
Hypothesis-generating gut–mesentery–metabolic framework in mesenteric panniculitis (MP). Created in BioRender. Ispas, S. (2026) https://BioRender.com/6c7ruwg (accessed on 4 September 2026). Solid arrows represent relationships supported by indirect human evidence from non-MP populations; dotted arrows represent relationships supported by experimental or mechanistic evidence; dashed arrows toward mesenteric inflammatory susceptibility represent hypothesized MP-specific relationships for which direct causal evidence is lacking. Direct MP-specific evidence primarily concerns clinical, radiological, histopathological, and epidemiological features.

Resident immune cells and adipocytes may respond to microbial and inflammatory signals through innate immune pathways [2]. In MP, established histopathological features include fat necrosis, chronic inflammatory infiltrates, and fibrosis [23]. Current evidence does not demonstrate that these features arise directly from GV-induced dysbiosis or microbial translocation. The proposed convergence of GV, gut dysbiosis, vascular dysfunction, and mesenteric inflammatory susceptibility is summarized in Figure 1.

## 7. Discussion

### 7.1. Integration of Current Evidence

Gut dysbiosis, intestinal barrier dysfunction, and metabolic endotoxemia are interconnected mechanisms in metabolic inflammation [5]. Increased intestinal permeability may facilitate microbial-product translocation and contribute to systemic inflammatory responses [8]. Direct evidence that these mechanisms cause mesenteric panniculitis (MP) is currently lacking.

### 7.2. Translational Relevance of Microbiome Modulation

Clinical studies indicate that microbiome-targeted interventions may influence metabolic and inflammatory outcomes, although their effects are heterogeneous. In type 2 diabetes, a randomized, double-blind, placebo-controlled trial of synbiotic supplementation reported changes in fasting glucose, inflammatory markers, gut microbiota composition, bile acids, and GLP-1 concentrations [61]. By contrast, another randomized controlled trial of probiotic supplementation found no significant between-group improvements in several glycemic and inflammatory outcomes [66]. These findings indicate that the effects of microbiome-targeted interventions vary between populations with metabolic disorders. No randomized controlled trial has specifically evaluated microbiome-targeted therapy in patients with MP, and its relevance to MP remains unproven.

### 7.3. Limitations of the Current Evidence

Several limitations should be considered when interpreting the proposed gut–mesentery–metabolic framework. Direct MP-specific evidence linking gut dysbiosis, intestinal barrier dysfunction, metabolic endotoxemia, glycemic variability (GV), or vascular dysfunction to the pathogenesis of MP is currently limited or absent. Much of the mechanistic evidence discussed in this review derives from obesity, type 2 diabetes, inflammatory bowel disease, other metabolic conditions, or experimental models and therefore cannot be directly extrapolated to MP.

The terminology and diagnostic classification of MP and sclerosing mesenteritis remain heterogeneous across the literature, and radiologically detected MP does not necessarily correspond to clinically significant inflammatory disease [34]. Contemporary clinical guidance also emphasizes the need to interpret imaging findings in the appropriate clinical context and exclude relevant mimics [35].

The interpretation of surrogate biomarkers represents an additional limitation. Commercial zonulin assays may lack specificity for pre-haptoglobin-2 [57], while circulating LPS measurements are influenced by methodological and biological factors that complicate their interpretation as direct measures of biologically active endotoxemia [8].

Most available studies are cross-sectional, retrospective, or mechanistic, limiting the assessment of temporality and causality. Current evidence does not establish whether dysbiosis, altered intestinal permeability, metabolic endotoxemia, GV, or vascular dysfunction precedes MP, results from associated metabolic conditions, or represents a parallel phenomenon.

This review is narrative rather than systematic. Although a structured search strategy was used, no formal risk-of-bias assessment or quantitative synthesis was performed. Consequently, selection bias and heterogeneity in the underlying evidence cannot be excluded. Prospective MP-specific studies with standardized diagnostic criteria and longitudinal assessment are required to test the proposed relationships.

### 7.4. Future Directions and Precision Medicine Perspectives

Future MP-specific studies should determine whether microbiome-related features are associated with metabolic, inflammatory, and imaging phenotypes. Integrating microbial profiling, metabolomics, and clinical phenotyping may help characterize biological heterogeneity and identify candidate subgroups for prospective investigation [17]. Multi-omics approaches may further support the exploration of relationships between microbial, metabolic, and host factors [77]. Any potential diagnostic, prognostic, or therapeutic relevance of these approaches in MP requires prospective validation.

## 8. Conclusions

Mesenteric panniculitis (MP) is a heterogeneous condition whose pathogenesis remains incompletely understood. Current MP-specific evidence primarily supports clinical, radiological, histopathological, and epidemiological associations, whereas direct evidence linking MP to gut dysbiosis, intestinal barrier dysfunction, metabolic endotoxemia, glycemic variability (GV), or mesenteric vascular variants remains limited or absent.

In this narrative review, the gut–mesentery–metabolic axis is proposed as a hypothesis-generating framework rather than an established pathogenic pathway. Evidence derived from obesity, type 2 diabetes, inflammatory bowel disease, vascular biology, and experimental models provides biological plausibility for interactions between microbial, metabolic, vascular, and adipose tissue pathways; however, these observations cannot be directly extrapolated to MP.

Future prospective MP-specific studies should integrate standardized clinical and imaging phenotyping with microbiome profiling, validated assessments of intestinal barrier function and microbial translocation, metabolic characterization, GV assessment, and vascular evaluation. Such studies are needed to determine whether distinct biological phenotypes of MP exist and whether any components of the proposed framework have diagnostic or prognostic relevance.

At present, microbiome-targeted, metabolic, or vascular interventions should not be considered established treatments for MP based on the proposed framework.

## Figures and Tables

**Table 1:** Comparative features of healthy versus dysbiotic gut microbiota and their functional implications.

Feature/Function	Healthy Microbiota	Dysbiotic Microbiota	References
Microbial composition	High diversity and stability; dominated by *Firmicutes*, *Bacteroidetes*, *Actinobacteria*, and *Proteobacteria*	Loss of beneficial taxa; overgrowth of facultative anaerobes and pathobionts (e.g., *Enterobacteriaceae*, *Proteobacteria*)	[15,37,39]
Metabolic activity	Balanced production of short-chain fatty acids (SCFAs) (butyrate, acetate, propionate); regulation of bile acids; synthesis of vitamins	Reduced SCFA production (↓ butyrate); altered bile acid metabolism	[39,41,42]
Epithelial barrier function	Reinforces tight junctions, maintains mucus layer integrity, and preserves selective permeability	Disruption of tight junctions; ↑ intestinal permeability (“leaky gut”)	[38,41,43]
Immune modulation	Promotes immune tolerance and an anti-inflammatory immune profile	↑ pro-inflammatory signaling; ↑ Interleukin-6 (IL-6), Tumor necrosis factor alpha (TNF-α), and Interleukin-1 beta (IL-1β); ↓ immunoregulation	[3,15,44]
Systemic metabolic effects	Supports insulin sensitivity, lipid homeostasis, and glucose regulation	Associated with insulin resistance, metabolic syndrome, Metabolic dysfunction-associated fatty liver disease (MAFLD)/Metabolic dysfunction-associated steatotic liver disease (MASLD), and type 2 diabetes	[39,41]
Interactions with intestinal and adjacent adipose tissues	Maintains local immune homeostasis; limited aberrant inflammatory signaling	May promote inflammatory signaling through intestinal barrier disruption, microbial translocation, and interactions between the intestine and adjacent adipose tissues	[38,40,45,46]

↑ indicates increased; ↓ indicates decreased.

**Table 2:** Representative randomized controlled trials of microbiome-targeted interventions in metabolic disorders: indirect evidence relevant to the proposed framework.

Study/Population	Intervention	Duration	Main Findings	Reference
Adults with obesity	Synbiotic: *Bifidobacterium animalis subsp. lactis* MN-Gup + GOS + XOS vs. placebo	12 weeks	Reduced body fat percentage, waist circumference, and LDL-C; associated changes in gut microbiota, bile acids, and gut hormones	[65]
Adults with type 2 diabetes	Synbiotic vs. probiotic alone vs. placebo	12 weeks	Synbiotic reduced fasting glucose and modified inflammatory markers, oxidative stress, gut microbiota, bile acids, and GLP-1; not all glycemic outcomes differed significantly between groups	[61]
Adults with type 2 diabetes	High-dose probiotic vs. placebo	12 weeks	No significant between-group improvements in HbA1c, glucose, insulin, LDL-C, triglycerides, or hs-CRP	[66]
Adults with prediabetes or type 2 diabetes	Synbiotic EDC-HHA01 vs. placebo	6 months	Greater improvements in HbA1c, fasting insulin, insulin resistance indices, and endotoxemia markers in prediabetes; effects were more modest in established type 2 diabetes	[64]

## Data Availability

No new data were generated or analyzed in this review. Data sharing is not applicable to this article.

## Supplementary Materials

The following supporting information can be downloaded at: https://www.mdpi.com/article/10.3390/biomedicines14092017/s1, Table S1. PubMed search strategies by thematic block; Table S2. Web of Science search strategies by thematic block; Table S3. Scopus search strategies by thematic block; Table S4. Inclusion and exclusion criteria.

## Abbreviations

The following abbreviations are used in this manuscript:

AMI	Acute mesenteric ischemia
CagA	Cytotoxin-associated gene A
CGM	Continuous glucose monitoring
CMI	Chronic mesenteric ischemia
CT	Computed tomography
FDG	Fluorodeoxyglucose
GIP	Glucose-dependent insulinotropic polypeptide
GLP-1	Glucagon-like peptide-1
GOS	Galactooligosaccharides
GV	Glycemic variability
HbA1c	Glycated hemoglobin A1c
HIF-1α	Hypoxia-inducible factor 1-alpha
hs-CRP	High-sensitivity C-reactive protein
IFN-γ	Interferon gamma
IL-1β	Interleukin-1 beta
IL-6	Interleukin-6
IL-17	Interleukin-17
LDL-C	Low-density lipoprotein cholesterol
LPS	Lipopolysaccharide
MAFLD	Metabolic dysfunction-associated fatty liver disease
MAGE	Mean amplitude of glycemic excursions
MAPK	Mitogen-activated protein kinase
MASLD	Metabolic dysfunction-associated steatotic liver disease
MCP-1	Monocyte chemoattractant protein-1
MP	Mesenteric panniculitis
MRI	Magnetic resonance imaging
NF-κB	Nuclear factor kappa B
PET/CT	Positron emission tomography/computed tomography
ROS	Reactive oxygen species
SCFAs	Short-chain fatty acids
SGLT2	Sodium–glucose cotransporter 2
SMA	Superior mesenteric artery
T2D	Type 2 diabetes
TIR	Time in Range
TLR4	Toll-like receptor 4
TNF-α	Tumor necrosis factor alpha
VacA	Vacuolating cytotoxin A
VCAM-1	Vascular cell adhesion molecule-1
XOS	Xylooligosaccharides
ZO-1	Zonula occludens-1
