# Supplementary material for: Mesenteric Panniculitis and the Gut–Mesentery–Metabolic Axis: A Hypothesis-Generating Narrative Review
Source: Biomedicines. 2026 Sep 8;14(9):2017. doi: 10.3390/biomedicines14092017 (PMC13604370; doi:10.3390/biomedicines14092017)
Supplement: Supplementary file 1 [file biomedicines-14-02017-s001.zip › Supplementary Table S4.pdf]

Supplementary Table S4. Inclusion and exclusion criteria

Category	Inclusion criteria	Exclusion criteria
**Topic relevance**	Studies addressing mesenteric panniculitis, sclerosing mesenteritis, mesenteric inflammation, mesenteric adipose tissue, gut dysbiosis, intestinal barrier dysfunction, microbial translocation, metabolic endotoxemia, glycemic variability, mesenteric vascular anatomy, vascular dysfunction, or regional perfusion.	Studies without a clear relationship to mesenteric panniculitis, mesenteric inflammation, gut microbiota-related mechanisms, vascular perfusion, glycemic variability, or metabolic inflammation.
**Study type**	Human studies, animal studies, experimental and translational studies, anatomical and imaging studies, clinical and observational studies, randomized controlled trials, narrative reviews, systematic reviews, and meta-analyses.	Conference abstracts without accessible full text, letters, comments, editorials without relevant mechanistic or clinical discussion, and opinion pieces.
**Clinical and pathological relevance**	Studies reporting clinical manifestations, radiological findings, histopathological features, differential diagnosis, natural history, or management of mesenteric panniculitis.	Studies without relevant clinical, pathological, imaging, or diagnostic information concerning mesenteric panniculitis or related mesenteric inflammatory disorders.
**Mechanistic relevance**	Studies addressing gut dysbiosis, intestinal permeability, microbial translocation, lipopolysaccharide-mediated signaling, TLR4-related pathways, metabolic endotoxemia, adipose-tissue inflammation, oxidative stress, endothelial dysfunction, ischemia–reperfusion, or glycemic variability.	Studies mentioning these mechanisms only superficially, without mechanistic, translational, or clinical relevance to the proposed gut–mesentery–metabolic axis.
**Microbiome–metabolic relevance**	Studies examining microbiota composition, microbial metabolites, intestinal barrier function, microbiome–host interactions, or systemic metabolic and inflammatory consequences.	Studies focused on microbiota without relevance to metabolic, inflammatory, intestinal barrier, or mesenteric mechanisms.
**Helicobacter pylori relevance**	Studies on Helicobacter pylori were included when they addressed gut microbiota alterations, dysbiosis, systemic inflammation, insulin resistance, metabolic syndrome, endothelial dysfunction, eradication-related microbiota changes, or other microbiome–metabolic	Studies on H. pylori without relevant microbiome, inflammatory, metabolic, vascular, or mechanistic information.

	mechanisms relevant to the review.	
**Therapeutic relevance**	Studies examining probiotics, prebiotics, synbiotics, postbiotics, short-chain fatty acids, microbiome-targeted interventions, glucose-lowering therapies, anti-inflammatory therapies, or vascular interventions relevant to mechanisms discussed in the review.	Studies focused on interventions unrelated to microbiome modulation, metabolic inflammation, vascular dysfunction, glycemic variability, or mesenteric inflammatory mechanisms.
**Population/model**	Studies involving humans, animal models, in vitro models, or experimental systems relevant to mesenteric inflammation, adipose-tissue biology, metabolic disease, vascular dysfunction, or gut microbiota-related mechanisms.	Studies conducted in populations or experimental models without relevance to the clinical or mechanistic framework of the review.
**Language**	Articles published in English.	Articles not published in English.
**Publication period**	Articles published between 2018 and 2026 were prioritized. Earlier seminal studies were included when they provided important anatomical, histopathological, radiological, mechanistic, translational, or clinical information relevant to mesenteric panniculitis and the proposed gut–mesentery–metabolic axis.	Older articles were excluded when they were not foundational, mechanistically relevant, or when the same evidence was adequately represented by more recent publications.
**Availability**	Full-text articles available for assessment.	Articles for which the full text was not available.
