# Supplementary material for: Mesenteric Panniculitis and the Gut–Mesentery–Metabolic Axis: A Hypothesis-Generating Narrative Review
Source: Biomedicines. 2026 Sep 8;14(9):2017. doi: 10.3390/biomedicines14092017 (PMC13604370; doi:10.3390/biomedicines14092017)
Supplement: Supplementary file 1 [file biomedicines-14-02017-s001.zip › Supplementary Tables S1, S2, S3.pdf]

Supplementary Table S1. PubMed search strategies by thematic block

Block	Search strategy	Records	Date filter	Thematic focus
A1	("mesenteric panniculitis"[tiab] OR "sclerosing mesenteritis"[tiab] OR "retractile mesenteritis"[tiab] OR "mesenteric lipodystrophy"[tiab] OR "misty mesentery"[tiab] OR "mesenteric fat necrosis"[tiab] OR "Panniculitis, Peritoneal"[MeSH]) AND (diagnosis[tiab] OR "differential diagnosis"[tiab] OR "desmoid fibromatosis"[tiab] OR "desmoid tumor"[tiab] OR "IgG4-related disease"[tiab] OR "computed tomography"[tiab] OR prevalence[tiab] OR treatment[tiab] OR outcome[tiab])	367 (unrestricted) / 156 (2018–2026)	English; None (primary) / 2018–2026 (secondary)	MP clinical/diagnostic core
A2a	("intestinal permeability"[tiab] OR "intestinal barrier"[tiab] OR "leaky gut"[tiab] OR "tight junction"[tiab] OR zonulin[tiab]) AND ("gut microbiota"[tiab] OR dysbiosis[tiab] OR "gut microbiome"[tiab]) AND (intestinal[ti] OR gut[ti]) AND 2018:2026[dp]	7,207	English; 2018–2026	Intestinal barrier/permeability
A2b	("metabolic endotoxemia"[tiab] OR lipopolysaccharide*[tiab] OR "microbial translocation"[tiab]) AND ("insulin resistance"[tiab] OR "metabolic syndrome"[tiab] OR obesity[tiab] OR "type 2 diabetes"[tiab]) AND 2018:2026[dp]	1,999	English; 2018–2026	Metabolic endotoxemia
A2c	("short chain fatty acid*"[tiab] OR butyrate[tiab] OR "bile acid*"[tiab] OR "microbiota metabolites"[tiab]) AND	529	English; 2018–2026	Microbial metabolites

	("intestinal barrier"[ti] OR "glucose metabolism"[ti]) AND 2018:2026[dp]			
A2d	(probiotic*[tiab] OR prebiotic*[tiab] OR synbiotic*[tiab] OR postbiotic*[tiab]) AND ("type 2 diabetes"[tiab] OR obesity[tiab] OR "glycemic control"[tiab]) AND ("randomized controlled trial"[tiab] OR "randomized controlled trial"[pt])	367 (unrestricted, RCT-filtered only)/ 280 (2018–2026)	English; None (primary) / 2018–2026 (secondary)	Microbiome-targeted interventions / RCTs
A2e	("mesenteric adipose tissue"[tiab] OR "visceral adiposity"[tiab] OR adipokine*[tiab]) AND ("inflammatory bowel disease"[tiab] OR "Crohn"[tiab] OR "intestinal inflammation"[tiab]) AND 2018:2026[dp]	179	English; 2018–2026	Mesenteric adipose tissue / IBD
A2f	("gut microbiota"[tiab] OR "gut microbiome"[tiab] OR dysbiosis[tiab]) AND (NAFLD[tiab] OR MAFLD[tiab] OR MASLD[tiab] OR "non-alcoholic fatty liver"[tiab] OR "metabolic dysfunction-associated fatty liver disease"[tiab] OR "metabolic dysfunction-associated steatotic liver disease"[tiab]) AND 2018:2026[dp]	2,576	English; 2018–2026	Microbiota–NAFLD/MAFLD/MASLD
A3a	("mesenteric vascular anatomy"[tiab] OR "mesenteric anatomy"[tiab] OR "superior mesenteric artery"[tiab] OR "inferior mesenteric artery"[tiab] OR "celiac trunk"[tiab] OR "coeliac trunk"[tiab] OR "marginal artery of Drummond"[tiab] OR "arc of Buhler"[tiab] OR "visceral adipose tissue"[tiab] OR "adipose tissue microcirculation"[tiab] OR "adipose tissue	2,214	English; 2018–2026	Mesenteric vascular anatomy / visceral adipose biology

	hypoxia”[tiab]) AND (anatom*[tiab] OR variant*[tiab] OR pathophysiology[tiab] OR microcirculation[tiab] OR hypoxia[tiab] OR “insulin resistance”[tiab]) AND 2018:2026[dp]			
A3b	(“chronic mesenteric ischemia”[tiab] OR “abdominal angina”[tiab] OR “mesenteric ischemia-reperfusion”[tiab] OR “intestinal ischemia-reperfusion”[tiab])	2,281 (unrestricted) / 853 (2018–2026)	English; None (primary) / 2018–2026 (secondary)	Mesenteric ischemia / reperfusion
A4a	(“glycemic variability”[tiab] OR “glycaemic variability”[tiab] OR “glucose variability”[tiab] OR “glucose fluctuations”[tiab] OR “diabetic cardiac autonomic neuropathy”[tiab]) AND (“diabetic complications”[tiab] OR “vascular dysfunction”[tiab] OR “cardiovascular disease”[tiab] OR “oxidative stress”[tiab]) AND 2018:2026[dp]	316	English; 2018–2026	Glycemic variability
A4b	(“continuous glucose monitoring”[tiab] OR “GLP-1 receptor agonist*”[tiab] OR “GIP receptor agonist*”[tiab] OR adiponectin[tiab] OR resistin[tiab]) AND (“cardiovascular risk”[tiab] OR “cardiometabolic risk”[tiab] OR “metabolic syndrome”[tiab] OR “metabolic inflammation”[tiab] OR “endoplasmic reticulum stress”[tiab]) AND 2018:2026[dp]	2,334	English; 2018–2026	Cardiometabolic risk markers
A5	(“Helicobacter pylori”[Title] OR “H. pylori”[Title]) AND (“gut microbiota”[Title] OR “eradication”[Title] OR “gastric carcinogenesis”[Title] OR	1,789	English; 2018–2026	H. pylori metabolic/vascular context

	“metabolic syndrome”[Title] OR “vascular aging”[Title] OR “endothelial dysfunction”[Title] OR “arterial stiffness”[Title]) AND 2018:2026[dp]			
A1 AND (A2a OR A2b OR A2c OR A2d OR A2e OR A2f)	(("mesenteric panniculitis"[tiab] OR "sclerosing mesenteritis"[tiab] OR "retractile mesenteritis"[tiab] OR "mesenteric lipodystrophy"[tiab] OR "misty mesentery"[tiab] OR "mesenteric fat necrosis"[tiab] OR "Panniculitis, Peritoneal"[MeSH]) AND (diagnosis[tiab] OR "differential diagnosis"[tiab] OR "desmoid fibromatosis"[tiab] OR "desmoid tumor"[tiab] OR "IgG4-related disease"[tiab] OR "computed tomography"[tiab] OR prevalence[tiab] OR treatment[tiab] OR outcome[tiab])) AND ((("intestinal permeability"[tiab] OR "intestinal barrier"[tiab] OR "leaky gut"[tiab] OR "tight junction"[tiab] OR zonulin[tiab]) AND ("gut microbiota"[tiab] OR dysbiosis[tiab] OR "gut microbiome"[tiab]) AND (intestinal[ti] OR gut[ti])) OR (("metabolic endotoxemia"[tiab] OR lipopolysaccharide*[tiab] OR "microbial translocation"[tiab]) AND ("insulin resistance"[tiab] OR "metabolic syndrome"[tiab] OR obesity[tiab] OR "type 2 diabetes"[tiab])) OR (("short chain fatty acid*"[tiab] OR butyrate[tiab] OR "bile acid*"[tiab] OR	1	English; 2018–2026	Limited direct co-occurrence between mesenteric panniculitis and gut microbiota/barrier/metabolic mechanisms; used as a sensitivity search for MP-specific evidence.

	"microbiota metabolites"[tiab]) AND ("intestinal barrier"[ti] OR "glucose metabolism"[ti])) OR ((probiotic*[tiab] OR prebiotic*[tiab] OR synbiotic*[tiab] OR postbiotic*[tiab]) AND ("type 2 diabetes"[tiab] OR obesity[tiab] OR "glycemic control"[tiab]) AND ("randomized controlled trial"[tiab] OR "randomized controlled trial"[pt])) OR (("mesenteric adipose tissue"[tiab] OR "visceral adiposity"[tiab] OR adipokine*[tiab]) AND ("inflammatory bowel disease"[tiab] OR "Crohn"[tiab] OR "intestinal inflammation"[tiab])) OR (("gut microbiota"[tiab] OR "gut microbiome"[tiab] OR dysbiosis[tiab]) AND (NAFLD[tiab] OR MAFLD[tiab] OR MASLD[tiab] OR "non-alcoholic fatty liver"[tiab] OR "metabolic dysfunction-associated fatty liver disease"[tiab] OR "metabolic dysfunction-associated steatotic liver disease"[tiab])))			
A1 AND (A3a OR A3b)	(("mesenteric panniculitis"[tiab] OR "sclerosing mesenteritis"[tiab] OR "retractile mesenteritis"[tiab] OR "mesenteric lipodystrophy"[tiab] OR "misty mesentery"[tiab] OR "mesenteric fat necrosis"[tiab] OR "Panniculitis, Peritoneal"[MeSH]) AND (diagnosis[tiab] OR "differential diagnosis"[tiab] OR "desmoid fibromatosis"[tiab] OR	1	English; 2018–2026	Limited direct co-occurrence identified between mesenteric panniculitis and vascular/perfusion-related terms; used as a sensitivity search for MP-specific evidence.

	"desmoid tumor"[tiab] OR "IgG4-related disease"[tiab] OR "computed tomography"[tiab] OR prevalence[tiab] OR treatment[tiab] OR outcome[tiab])) AND ((("mesenteric vascular anatomy"[tiab] OR "mesenteric anatomy"[tiab] OR "superior mesenteric artery"[tiab] OR "inferior mesenteric artery"[tiab] OR "celiac trunk"[tiab] OR "coeliac trunk"[tiab] OR "marginal artery of Drummond"[tiab] OR "arc of Buhler"[tiab] OR "visceral adipose tissue"[tiab] OR "adipose tissue microcirculation"[tiab] OR "adipose tissue hypoxia"[tiab]) AND (anatom*[tiab] OR variant*[tiab] OR pathophysiology[tiab] OR microcirculation[tiab] OR hypoxia[tiab] OR "insulin resistance"[tiab])) OR ("chronic mesenteric ischemia"[tiab] OR "abdominal angina"[tiab] OR "mesenteric ischemia-reperfusion"[tiab] OR "intestinal ischemia-reperfusion"[tiab]))			
A1 AND (A4a OR A4b)	(("mesenteric panniculitis"[tiab] OR "sclerosing mesenteritis"[tiab] OR "retractile mesenteritis"[tiab] OR "mesenteric lipodystrophy"[tiab] OR "misty mesentery"[tiab] OR "mesenteric fat necrosis"[tiab] OR "Panniculitis, Peritoneal"[MeSH]) AND (diagnosis[tiab] OR "differential diagnosis"[tiab] OR "desmoid fibromatosis"[tiab] OR	0	English; 2018–2026	No direct co-occurrence identified between mesenteric panniculitis and glycemic variability/cardiometabolic mechanisms.

	"desmoid tumor"[tiab] OR "IgG4-related disease"[tiab] OR "computed tomography"[tiab] OR prevalence[tiab] OR treatment[tiab] OR outcome[tiab])) AND ((("glycemic variability"[tiab] OR "glycaemic variability"[tiab] OR "glucose variability"[tiab] OR "glucose fluctuations"[tiab] OR "diabetic cardiac autonomic neuropathy"[tiab]) AND ("diabetic complications"[tiab] OR "vascular dysfunction"[tiab] OR "cardiovascular disease"[tiab] OR "oxidative stress"[tiab])) OR (("continuous glucose monitoring"[tiab] OR "GLP-1 receptor agonist*"[tiab] OR "GIP receptor agonist*"[tiab] OR adiponectin[tiab] OR resistin[tiab]) AND ("cardiovascular risk"[tiab] OR "cardiometabolic risk"[tiab] OR "metabolic syndrome"[tiab] OR "metabolic inflammation"[tiab] OR "endoplasmic reticulum stress"[tiab])))			
A1 AND A5	(("mesenteric panniculitis"[tiab] OR "sclerosing mesenteritis"[tiab] OR "retractile mesenteritis"[tiab] OR "mesenteric lipodystrophy"[tiab] OR "misty mesentery"[tiab] OR "mesenteric fat necrosis"[tiab] OR "Panniculitis, Peritoneal"[MeSH]) AND	0	English; 2018–2026	No direct co-occurrence identified between mesenteric panniculitis and the H. pylori-related mechanistic domain.

(diagnosis[tiab] OR "differential diagnosis"[tiab] OR "desmoid fibromatosis"[tiab] OR "desmoid tumor"[tiab] OR "IgG4-related disease"[tiab] OR "computed tomography"[tiab] OR prevalence[tiab] OR treatment[tiab] OR outcome[tiab])
)
AND
(
("Helicobacter pylori"[Title] OR "H. pylori"[Title])
AND
("gut microbiota"[Title] OR eradication[Title] OR "gastric carcinogenesis"[Title] OR "metabolic syndrome"[Title] OR "vascular aging"[Title] OR "endothelial dysfunction"[Title] OR "arterial stiffness"[Title])
)

Supplementary Table S2. Web of Science search strategies by thematic block

Block	Search strategy	Records	Date filter	Thematic focus
B1	TS=("mesenteric panniculitis" OR "sclerosing mesenteritis" OR "retractile mesenteritis" OR "mesenteric lipodystrophy" OR "misty mesentery" OR "mesenteric fat necrosis" OR "Panniculitis, Peritoneal") AND TS=(diagnosis OR "differential diagnosis" OR "desmoid fibromatosis" OR "desmoid tumor" OR "IgG4-related disease" OR "computed tomography" OR prevalence OR treatment OR outcome)	175	English; 2018–2026	MP clinical/diagnostic core

B2a	TS=("intestinal permeability" OR "intestinal barrier" OR "leaky gut" OR "tight junction" OR zonulin) AND TS=("gut microbiota" OR dysbiosis OR "gut microbiome") AND TS=(intestinal OR gut)	12,811	English; 2018–2026	Intestinal barrier/permeability
B2b	TS=("metabolic endotoxemia" OR lipopolysaccharide* OR "microbial translocation") AND TS=("insulin resistance" OR "metabolic syndrome" OR obesity OR "type 2 diabetes")	3,057	English; 2018–2026	Metabolic endotoxemia
B2c	TS=("short chain fatty acid*" OR butyrate OR "bile acid*" OR "microbiota metabolites") AND TS=("intestinal barrier" OR "glucose metabolism")	4,438	English; 2018–2026	Microbial metabolites
B2d	TS=(probiotic* OR prebiotic* OR synbiotic* OR postbiotic*) AND TS=("type 2 diabetes" OR obesity OR "glycemic control") AND TS=("randomized controlled trial")	148	English; 2018–2026	Microbiome-targeted interventions / RCTs
B2e	TS=("mesenteric adipose tissue" OR "visceral adiposity" OR adipokine*) AND TS=("inflammatory bowel disease" OR Crohn* OR "intestinal inflammation")	244	English; 2018–2026	Mesenteric adipose tissue / IBD
B2f	TS=("gut microbiota" OR "gut microbiome" OR dysbiosis) AND TS=(NAFLD OR MAFLD OR MASLD OR "non-alcoholic fatty liver" OR "metabolic dysfunction-associated fatty liver disease" OR "metabolic dysfunction-associated steatotic liver disease")	3,389	English; 2018–2026	Microbiota–NAFLD/MAFLD/MASLD

B3a	TS=("mesenteric vascular anatomy" OR "mesenteric anatomy" OR "superior mesenteric artery" OR "inferior mesenteric artery" OR "celiac trunk" OR "coeliac trunk" OR "marginal artery of Drummond" OR "arc of Buhler" OR "visceral adipose tissue" OR "adipose tissue microcirculation" OR "adipose tissue hypoxia") AND TS=(anatom* OR variant* OR pathophysiology OR microcirculation OR hypoxia OR "insulin resistance")	3,120	English; 2018–2026	Mesenteric vascular anatomy / visceral adipose biology
B3b	TS=("chronic mesenteric ischemia" OR "abdominal angina" OR "mesenteric ischemia-reperfusion" OR "intestinal ischemia-reperfusion")	3,053	English; 2018–2026	Mesenteric ischemia / reperfusion
B4a	TS=("glycemic variability" OR "glycaemic variability" OR "glucose variability" OR "glucose fluctuations" OR "diabetic cardiac autonomic neuropathy") AND TS=("diabetic complications" OR "vascular dysfunction" OR "cardiovascular disease" OR "oxidative stress")	763	English; 2018–2026	Glycemic variability
B4b	TS=("continuous glucose monitoring" OR "GLP-1 receptor agonist*" OR "GIP receptor agonist*" OR adiponectin OR resistin) AND TS=("cardiovascular risk" OR "cardiometabolic risk" OR "metabolic syndrome" OR "metabolic	4,047	English; 2018–2026	Cardiometabolic risk markers

	inflammation" OR "endoplasmic reticulum stress")			
B5	TS=("Helicobacter pylori" OR "H. pylori") AND TS=("gut microbiota" OR eradication OR "gastric carcinogenesis" OR "metabolic syndrome" OR "vascular aging" OR "endothelial dysfunction" OR "arterial stiffness")	7,473	English; 2018–2026	H. pylori metabolic/vascular context
B1 AND (B2a OR B2b OR B2c OR B2d OR B2e OR B2f)	TS=("mesenteric panniculitis" OR "sclerosing mesenteritis" OR "retractile mesenteritis" OR "mesenteric lipodystrophy" OR "misty mesentery" OR "mesenteric fat necrosis" OR "peritoneal panniculitis") AND TS=(diagnosis OR "differential diagnosis" OR "desmoid fibromatosis" OR "desmoid tumor" OR "IgG4-related disease" OR "computed tomography" OR prevalence OR treatment OR outcome) AND (TS=("intestinal permeability" OR "intestinal barrier" OR "leaky gut" OR "tight junction" OR zonulin) AND TS=("gut microbiota" OR dysbiosis OR "gut microbiome") AND TS=(intestinal OR gut) OR TS=("metabolic endotoxemia" OR lipopolysaccharide* OR "microbial translocation") AND TS=("insulin resistance" OR "metabolic syndrome" OR obesity OR "type 2 diabetes") OR TS=("short chain fatty acid*" OR butyrate OR "bile acid*" OR	1	English; 2018–2026	Limited direct co-occurrence between mesenteric panniculitis and gut microbiota/barrier/metabolic mechanisms; used as a sensitivity search for MP-specific evidence.

	"microbiota metabolites") AND TS=("intestinal barrier" OR "glucose metabolism") OR TS=(probiotic* OR prebiotic* OR synbiotic* OR postbiotic*) AND TS=("type 2 diabetes" OR obesity OR "glycemic control") AND TS=("randomized controlled trial") OR TS=("mesenteric adipose tissue" OR "visceral adiposity" OR adipokine*) AND TS=("inflammatory bowel disease" OR Crohn* OR "intestinal inflammation") OR TS=("gut microbiota" OR "gut microbiome" OR dysbiosis) AND TS=(NAFLD OR MAFLD OR MASLD OR "non-alcoholic fatty liver" OR "metabolic dysfunction-associated fatty liver disease" OR "metabolic dysfunction-associated steatotic liver disease"))			
B1 AND (B3a OR B3b)	TS=("mesenteric panniculitis" OR "sclerosing mesenteritis" OR "retractile mesenteritis" OR "mesenteric lipodystrophy" OR "misty mesentery" OR "mesenteric fat necrosis" OR "peritoneal panniculitis") AND TS=(diagnosis OR "differential diagnosis" OR "desmoid fibromatosis" OR "desmoid tumor" OR "IgG4-related disease" OR "computed tomography" OR prevalence OR treatment OR outcome) AND ((TS=("mesenteric vascular anatomy" OR "mesenteric anatomy"	5	English; 2018–2026	Limited direct co-occurrence identified between mesenteric panniculitis and vascular/perfusion-related terms; used as a sensitivity search for MP-specific evidence.

	OR "superior mesenteric artery" OR "inferior mesenteric artery" OR "celiac trunk" OR "coeliac trunk" OR "marginal artery of Drummond" OR "arc of Buhler" OR "visceral adipose tissue" OR "adipose tissue microcirculation" OR "adipose tissue hypoxia") AND TS=(anatom* OR variant* OR pathophysiology OR microcirculation OR hypoxia OR "insulin resistance")) OR TS=("chronic mesenteric ischemia" OR "abdominal angina" OR "mesenteric ischemia-reperfusion" OR "intestinal ischemia-reperfusion"))			
B1 AND (B4a OR B4b)	TS=("mesenteric panniculitis" OR "sclerosing mesenteritis" OR "retractile mesenteritis" OR "mesenteric lipodystrophy" OR "misty mesentery" OR "mesenteric fat necrosis" OR "peritoneal panniculitis") AND TS=(diagnosis OR "differential diagnosis" OR "desmoid fibromatosis" OR "desmoid tumor" OR "IgG4-related disease" OR "computed tomography" OR prevalence OR treatment OR outcome) AND ((TS=("glycemic variability" OR "glycaemic variability" OR "glucose variability" OR "glucose fluctuations" OR "diabetic cardiac autonomic neuropathy") AND TS=("diabetic	0	English; 2018–2026	No direct co-occurrence identified between mesenteric panniculitis and glycemic variability/cardiometabolic mechanisms.

	complications" OR "vascular dysfunction" OR "cardiovascular disease" OR "oxidative stress")) OR (TS=("continuous glucose monitoring" OR "GLP-1 receptor agonist*" OR "GIP receptor agonist*" OR adiponectin OR resistin) AND TS=("cardiovascular risk" OR "cardiometabolic risk" OR "metabolic syndrome" OR "metabolic inflammation" OR "endoplasmic reticulum stress")))			
B1 AND B5	TS=("mesenteric panniculitis" OR "sclerosing mesenteritis" OR "retractile mesenteritis" OR "mesenteric lipodystrophy" OR "misty mesentery" OR "mesenteric fat necrosis" OR "peritoneal panniculitis") AND TS=(diagnosis OR "differential diagnosis" OR "desmoid fibromatosis" OR "desmoid tumor" OR "IgG4-related disease" OR "computed tomography" OR prevalence OR treatment OR outcome) AND TS=("Helicobacter pylori" OR "H. pylori") AND TS=("gut microbiota" OR eradication OR "gastric carcinogenesis" OR "metabolic syndrome" OR "vascular aging" OR "endothelial dysfunction" OR "arterial stiffness")	0	English; 2018–2026	No direct co-occurrence identified between mesenteric panniculitis and H. pylori-related mechanistic terms.

Supplementary Table S3. Scopus search strategies by thematic block

Block	Search strategy	Records	Date filter	Thematic focus
C1	TITLE-ABS-KEY("mesenteric panniculitis" OR "sclerosing mesenteritis" OR "retractile mesenteritis" OR "mesenteric lipodystrophy" OR "misty mesentery" OR "mesenteric fat necrosis" OR "Panniculitis, Peritoneal") AND TITLE-ABS-KEY(diagnosis OR "differential diagnosis" OR "desmoid fibromatosis" OR "desmoid tumor" OR "IgG4-related disease" OR "computed tomography" OR prevalence OR treatment OR outcome) AND LANGUAGE(english)	251	English; 2018–2026	MP clinical/diagnostic core
C2a	TITLE-ABS-KEY("intestinal permeability" OR "intestinal barrier" OR "leaky gut" OR "tight junction" OR zonulin) AND TITLE-ABS-KEY("gut microbiota" OR dysbiosis OR "gut microbiome") AND (TITLE(intestinal) OR TITLE(gut)) AND PUBYEAR > 2017 AND PUBYEAR < 2027 AND LANGUAGE(english) AND PUBYEAR > 2017 AND PUBYEAR < 2027	8,554	English; 2018–2026	Intestinal barrier/permeability
C2b	TITLE-ABS-KEY("metabolic endotoxemia" OR lipopolysaccharide* OR "microbial translocation") AND TITLE-ABS-KEY("insulin resistance" OR "metabolic syndrome" OR obesity OR "type 2 diabetes") AND PUBYEAR > 2017 AND PUBYEAR < 2027 AND LANGUAGE(english)	3,628	English; 2018–2026	Metabolic endotoxemia
C2c	TITLE-ABS-KEY("short chain fatty acid*" OR butyrate OR "bile acid*"	668	English; 2018–2026	Microbial metabolites

	OR "microbiota metabolites") AND (TITLE("intestinal barrier") OR TITLE("glucose metabolism")) AND PUBYEAR > 2017 AND PUBYEAR < 2027 AND LANGUAGE(english)			
C2d	TITLE-ABS-KEY(probiotic* OR prebiotic* OR synbiotic* OR postbiotic*) AND TITLE-ABS-KEY("type 2 diabetes" OR obesity OR "glycemic control") AND TITLE-ABS-KEY("randomized controlled trial") AND LANGUAGE(english) AND PUBYEAR > 2017 AND PUBYEAR < 2027	1,292	English; 2018–2026	Microbiome-targeted interventions / RCTs
C2e	TITLE-ABS-KEY("mesenteric adipose tissue" OR "visceral adiposity" OR adipokine*) AND TITLE-ABS-KEY("inflammatory bowel disease" OR Crohn* OR "intestinal inflammation") AND PUBYEAR > 2017 AND PUBYEAR < 2027 AND LANGUAGE(english)	214	English; 2018–2026	Mesenteric adipose tissue / IBD
C2f	TITLE-ABS-KEY("gut microbiota" OR "gut microbiome" OR dysbiosis) AND TITLE-ABS-KEY(NAFLD OR MAFLD OR MASLD OR "non-alcoholic fatty liver" OR "metabolic dysfunction-associated fatty liver disease" OR "metabolic dysfunction-associated steatotic liver disease") AND PUBYEAR > 2017 AND PUBYEAR < 2027 AND LANGUAGE(english) AND PUBYEAR > 2017 AND PUBYEAR < 2027	3,282	English; 2018–2026	Microbiota–NAFLD/MAFLD/MASLD
C3a	TITLE-ABS-KEY("mesenteric vascular anatomy" OR "mesenteric anatomy" OR "superior mesenteric artery" OR	4,021	English; 2018–2026	Mesenteric vascular anatomy / visceral adipose biology

	"inferior mesenteric artery" OR "celiac trunk" OR "coeliac trunk" OR "marginal artery of Drummond" OR "arc of Buhler" OR "visceral adipose tissue" OR "adipose tissue microcirculation" OR "adipose tissue hypoxia") AND TITLE-ABS-KEY(anatom* OR variant* OR pathophysiology OR microcirculation OR hypoxia OR "insulin resistance") AND PUBYEAR > 2017 AND PUBYEAR < 2027 AND LANGUAGE(english) AND PUBYEAR > 2017 AND PUBYEAR < 2027			
C3b	TITLE-ABS-KEY("chronic mesenteric ischemia" OR "abdominal angina" OR "mesenteric ischemia-reperfusion" OR "intestinal ischemia-reperfusion") AND LANGUAGE(english) AND PUBYEAR > 2017 AND PUBYEAR < 2027	1,077	English; 2018–2026	Mesenteric ischemia / reperfusion
C4a	TITLE-ABS-KEY("glycemic variability" OR "glycaemic variability" OR "glucose variability" OR "glucose fluctuations" OR "diabetic cardiac autonomic neuropathy") AND TITLE-ABS-KEY("diabetic complications" OR "vascular dysfunction" OR "cardiovascular disease" OR "oxidative stress") AND PUBYEAR > 2017 AND PUBYEAR < 2027 AND LANGUAGE(english) AND PUBYEAR > 2017 AND PUBYEAR < 2027	572	English; 2018–2026	Glycemic variability
C4b	TITLE-ABS-KEY("continuous glucose monitoring" OR "GLP-1 receptor agonist*" OR "GIP receptor agonist*" OR adiponectin OR	5,311	English; 2018–2026	Cardiometabolic risk markers

	resistin) AND TITLE-ABS-KEY("cardiovascular risk" OR "cardiometabolic risk" OR "metabolic syndrome" OR "metabolic inflammation" OR "endoplasmic reticulum stress") AND PUBYEAR > 2017 AND PUBYEAR < 2027 AND LANGUAGE(english) AND PUBYEAR > 2017 AND PUBYEAR < 2027			
C5	(TITLE("Helicobacter pylori") OR TITLE("H. pylori")) AND (TITLE("gut microbiota") OR TITLE(eradication) OR TITLE("gastric carcinogenesis") OR TITLE("metabolic syndrome") OR TITLE("vascular aging") OR TITLE("endothelial dysfunction") OR TITLE("arterial stiffness")) AND PUBYEAR > 2017 AND PUBYEAR < 2027 AND LANGUAGE(english)	1,869	English; 2018–2026	H. pylori metabolic/vascular context
C1 AND (C2a OR C2b OR C2c OR C2d OR C2e OR C2f)	(TITLE-ABS-KEY("mesenteric panniculitis" OR "sclerosing mesenteritis" OR "retractile mesenteritis" OR "mesenteric lipodystrophy" OR "misty mesentery" OR "mesenteric fat necrosis" OR "Panniculitis, Peritoneal") AND TITLE-ABS-KEY(diagnosis OR "differential diagnosis" OR "desmoid fibromatosis" OR "desmoid tumor" OR "IgG4-related disease" OR "computed tomography" OR prevalence OR treatment OR outcome)) AND (	1	English; 2018–2026	Limited direct co-occurrence between mesenteric panniculitis and gut microbiota/barrier/metabolic mechanisms; used as a sensitivity search for MP-specific evidence.

(
TITLE-ABS-
KEY("intestinal
permeability" OR
"intestinal barrier" OR
"leaky gut" OR "tight
junction" OR zonulin)
AND
TITLE-ABS-KEY("gut
microbiota" OR dysbiosis
OR "gut microbiome")
AND
(TITLE(intestinal) OR
TITLE(gut))
)
OR
(
TITLE-ABS-
KEY("metabolic
endotoxemia" OR
lipopolysaccharide* OR
"microbial translocation")
AND
TITLE-ABS-KEY("insulin
resistance" OR "metabolic
syndrome" OR obesity
OR "type 2 diabetes")
)
OR
(
TITLE-ABS-KEY("short
chain fatty acid*" OR
butyrate OR "bile acid*"
OR "microbiota
metabolites")
AND
(TITLE("intestinal
barrier") OR
TITLE("glucose
metabolism"))
)
OR
(
TITLE-ABS-
KEY(probiotic* OR
prebiotic* OR synbiotic*
OR postbiotic*)
AND
TITLE-ABS-KEY("type 2
diabetes" OR obesity OR
"glycemic control")
AND
TITLE-ABS-
KEY("randomized
controlled trial")
)
OR

	(TITLE-ABS-KEY("mesenteric adipose tissue" OR "visceral adiposity" OR adipokine*) AND TITLE-ABS-KEY("inflammatory bowel disease" OR Crohn* OR "intestinal inflammation")) OR (TITLE-ABS-KEY("gut microbiota" OR "gut microbiome" OR dysbiosis) AND TITLE-ABS-KEY(NAFLD OR MAFLD OR MASLD OR "non-alcoholic fatty liver" OR "metabolic dysfunction-associated fatty liver disease" OR "metabolic dysfunction-associated steatotic liver disease"))) AND PUBYEAR > 2017 AND PUBYEAR < 2027 AND LANGUAGE(english)			
C1 AND (C3a OR C3b)	(TITLE-ABS-KEY("mesenteric panniculitis" OR "sclerosing mesenteritis" OR "retractile mesenteritis" OR "mesenteric lipodystrophy" OR "misty mesentery" OR "mesenteric fat necrosis" OR "Panniculitis, Peritoneal") AND TITLE-ABS-KEY(diagnosis OR "differential diagnosis" OR "desmoid fibromatosis" OR "desmoid tumor" OR "IgG4-related disease" OR "computed tomography" OR prevalence OR treatment OR outcome)) AND ((TITLE-ABS-	4	English; 2018–2026	Limited direct co-occurrence identified between mesenteric panniculitis and vascular/perfusion-related terms; used as a sensitivity search for MP-specific evidence.

	KEY("mesenteric vascular anatomy" OR "mesenteric anatomy" OR "superior mesenteric artery" OR "inferior mesenteric artery" OR "celiac trunk" OR "coeliac trunk" OR "marginal artery of Drummond" OR "arc of Buhler" OR "visceral adipose tissue" OR "adipose tissue microcirculation" OR "adipose tissue hypoxia") AND TITLE-ABS-KEY(anatom* OR variant* OR pathophysiology OR microcirculation OR hypoxia OR "insulin resistance")) OR (TITLE-ABS-KEY("chronic mesenteric ischemia" OR "abdominal angina" OR "mesenteric ischemia-reperfusion" OR "intestinal ischemia-reperfusion"))) AND PUBYEAR > 2017 AND PUBYEAR < 2027 AND LANGUAGE(english)			
C1 AND (C4a OR C4b)	(TITLE-ABS-KEY("mesenteric panniculitis" OR "sclerosing mesenteritis" OR "retractile mesenteritis" OR "mesenteric lipodystrophy" OR "misty mesentery" OR "mesenteric fat necrosis" OR "Panniculitis, Peritoneal") AND TITLE-ABS-KEY(diagnosis OR "differential diagnosis" OR "desmoid fibromatosis" OR "desmoid tumor" OR "IgG4-related disease" OR "computed tomography" OR prevalence OR treatment OR outcome)) AND (	0	English; 2018–2026	No direct co-occurrence identified between mesenteric panniculitis and glycemic variability/cardiometabolic mechanisms.

	(<br>TITLE-ABS-KEY("glycemic variability" OR "glycaemic variability" OR "glucose variability" OR "glucose fluctuations" OR "diabetic cardiac autonomic neuropathy")<br>AND<br>TITLE-ABS-KEY("diabetic complications" OR "vascular dysfunction" OR "cardiovascular disease" OR "oxidative stress")<br>)<br>OR<br>(<br>TITLE-ABS-KEY("continuous glucose monitoring" OR "GLP-1 receptor agonist*" OR "GIP receptor agonist*" OR adiponectin OR resistin)<br>AND<br>TITLE-ABS-KEY("cardiovascular risk" OR "cardiometabolic risk" OR "metabolic syndrome" OR "metabolic inflammation" OR "endoplasmic reticulum stress")<br>)<br>)<br>AND PUBYEAR > 2017<br>AND PUBYEAR < 2027<br>AND<br>LANGUAGE(english)			
C1 AND C5	(<br>TITLE-ABS-KEY("mesenteric panniculitis" OR "sclerosing mesenteritis" OR "retractile mesenteritis" OR "mesenteric lipodystrophy" OR "misty mesentery" OR "mesenteric fat necrosis" OR "Panniculitis, Peritoneal")<br>AND<br>TITLE-ABS-KEY(diagnosis OR	0	English; 2018–2026	No direct co-occurrence identified between mesenteric panniculitis and H. pylori-related mechanistic terms.

"differential diagnosis"
OR "desmoid
fibromatosis" OR
"desmoid tumor" OR
"IgG4-related disease" OR
"computed tomography"
OR prevalence OR
treatment OR outcome)
)
AND
(
TITLE("Helicobacter
pylori" OR "H. pylori")
AND
TITLE("gut microbiota"
OR eradication OR
"gastric carcinogenesis"
OR "metabolic syndrome"
OR "vascular aging" OR
"endothelial dysfunction"
OR "arterial stiffness")
)
AND PUBYEAR > 2017
AND PUBYEAR < 2027
AND
LANGUAGE(english)
